# SARS-CoV-2 ORF7a Mutation Found in BF.5 and BF.7 Sublineages Impacts Its Functions

**DOI:** 10.3390/ijms25042351

**Published:** 2024-02-16

**Authors:** Uddhav Timilsina, Emily B. Ivey, Sean Duffy, Arnon Plianchaisuk, The Genotype to Phenotype Japan (G2P-Japan) Consortium, Jumpei Ito, Kei Sato, Spyridon Stavrou

**Affiliations:** 1 Department of Microbiology and Immunology, Jacobs School of Medicine and Biomedical Sciences, University at Buffalo, Buffalo, NY 14203, USA; uddhavti@buffalo.edu (U.T.); emilyive@buffalo.edu (E.B.I.); seanduff@buffalo.edu (S.D.); 2 Division of Systems Virology, Department of Microbiology and Immunology, The Institute of Medical Science, The University of Tokyo, Tokyo 108-8369, Japan; pcs-arnon@g.ecc.u-tokyo.ac.jp (A.P.); jampei0513@yahoo.co.jp (J.I.); keisato@g.ecc.u-tokyo.ac.jp (K.S.); 3 International Research Center for Infectious Diseases, The Institute of Medical Science, The University of Tokyo, Tokyo 108-8369, Japan; 4 Graduate School of Medicine, The University of Tokyo, Tokyo 113-8654, Japan; 5 Graduate School of Frontier Sciences, The University of Tokyo, Kashiwa 277-8581, Japan; 6 International Vaccine Design Center, The Institute of Medical Science, The University of Tokyo, Tokyo 108-8639, Japan; 7 Collaboration Unit for Infection, Joint Research Center for Human Retrovirus Infection, Kumamoto University, Kumamoto 860-0862, Japan; 8 CREST, Japan Science and Technology Agency, Kawaguchi 332-0012, Japan

**Keywords:** severe acute respiratory syndrome coronavirus 2, open reading frame 7a (ORF7a), mutation, type I interferon response, major histocompatibility complex I

## Abstract

A feature of the SARS-CoV-2 Omicron subvariants BF.5 and BF.7 that recently circulated mainly in China and Japan was the high prevalence of the ORF7a: H47Y mutation, in which the 47th residue of ORF7a has been mutated from a histidine (H) to a tyrosine (Y). Here, we evaluated the effect of this mutation on the three main functions ascribed to the SARS-CoV-2 ORF7a protein. Our findings show that H47Y mutation impairs the ability of SARS-CoV-2 ORF7a to antagonize the type I interferon (IFN-I) response and to downregulate major histocompatibility complex I (MHC-I) cell surface levels, but had no effect in its anti-SERINC5 function. Overall, our results suggest that the H47Y mutation of ORF7a affects important functions of this protein, resulting in changes in virus pathogenesis.

## 1. Introduction

Severe acute respiratory syndrome coronavirus 2 (SARS-CoV-2) has led to the global pandemic of coronavirus disease 2019 (COVID-19). The SARS-CoV-2 genome encodes four structural proteins (spike, S; membrane, M; envelope, E; and nucleocapsid, N) and a number of non-structural proteins (NSPs) involved in virus replication (NSP1 to NSP16) or modulation of host responses (open reading frame 3a (ORF3a), ORF3b, ORF6, ORF7a, ORF7b, ORF8, ORF9b, ORF9c, and ORF10) [1,2]. Over the course of the COVID-19 pandemic, the continued evolution of SARS-CoV-2 has led to the emergence of several variants (Alpha, Beta, Gamma, Delta, Epsilon, Eta, Ota, Kappa, Omicron, Zeta and Mu) [3,4,5]. The rise of SARS-CoV-2 variants of concern (VOCs) are characterized primarily by the emergence of mutations within the S protein. S mutations have led to altered virus biology facilitating evasion of vaccine- and infection-induced immunity, leading to enhanced transmissibility of SARS-CoV-2 VOCs [5,6,7]. In addition, emerging variants have mutations across other structural proteins (E, M, N), nonstructural proteins (NSP1, NSP3, NSP4, NSP5, NSP6, NSP12) and accessory proteins (ORF3a, ORF6, ORF7a, ORF8 or ORF10) [8,9]. It has been reported that new SARS-CoV-2 subvariants of Omicron—BF.7 (BA.5.2.1.7), initially prevalent in China, and BF.5, dominant in Japan in late 2022—have a unique non-synonymous mutation (H47Y) in ORF7a protein at position 47, where a histidine (H) has been mutated to a tyrosine (Y) [10,11].

SARS-CoV-2 ORF7a is a type 1 transmembrane protein with 121 amino acid residues, consisting of an N-terminal signaling region (residues 1–15), an immunoglobulin-like (Ig-like) ectodomain consisting of seven β-strands (strands A to G; residues 16–96), a transmembrane domain (TM) (97–116) and a C-terminal endoplasmic reticulum (ER)-retention motif (residues 117–121) [12]. Major functions ascribed to SARS-CoV-2 ORF7a during infection include impairing the antiviral effect of host factors, including serine incorporator 5 (SERINC5) [13] and bone marrow stromal antigen 2 (BST2)/tetherin [14], inhibiting the type I interferon (IFN-I) response [15,16], and downregulating the levels of major histocompatibility complex I (MHC-I) [17,18] on the cell surface. In addition, SARS-CoV-2 ORF7a has been associated with the induction of autophagy [19] and apoptosis [20] and upregulation of inflammatory responses [12,21].

SARS-CoV-2 ORF7a acts as a viral antagonist of SERINC5 by preventing its incorporation into nascent virions [13]. In addition, SARS-CoV-2 ORF7a interacts with spike and SERINC5, thereby counteracting SERINC5-mediated restriction of SARS-CoV-2 infectivity [13]. It has been shown that SERINC5, which inhibits virus–cell membrane fusion, interacts via its transmembrane domains with the transmembrane domain of ORF7a [13]. Furthermore, SARS-CoV-2 ORF7a is implicated in the evasion of the host immune response by antagonizing the type I interferon (IFN) response. SARS-CoV-2 ORF7a hijacks the host ubiquitin system to polyubiquitinate itself at K119 amino acid residue, thereby blocking the IFN-α-mediated phosphorylation of signal transducer and activator of transcription 2 (STAT2) [15,16]. Recent studies have determined that ORF7a interferes with the antigen presentation ability of host cells by interacting with the heavy chain of MHC-I, thereby disrupting the assembly of the MHC-I peptide-loading complex (PLC) in the endoplasmic reticulum (ER) and preventing export of peptide-loaded MHC-I complexes to the cell surface [17,18].

Global genomic surveillance during the COVID-19 pandemic has shown that deletion and substitution mutations in the SARS-CoV-2 ORF7a gene are frequent [22,23,24,25]. Though no study has determined the impact of these mutations in the clinical context, certain SARS-CoV-2 ORF7a deletion mutations are known to impair virus replication in vitro [22,26]. A SARS-CoV-2 strain with a truncated ORF7a (115 nucleotide deletion) was found to be defective in suppressing the host immune response [22]. A mutation (A105V) in the TM domain of SARS-CoV-2 ORF7a that improved its stability was associated with severe disease outcome among a group of Romanian COVID-19 patients [24]. Moreover, we and others have shown that in vitro deletion of the ORF7a gene reduces replication of synthetic recombinant SARS-CoV-2 virus in a cell type-specific manner [13,27], suggesting an important role of this protein in virus replication and pathogenesis.

Since late 2021, when the first Omicron variant (B.1.1.529) was reported, multiple Omicron subvariants (BA.1, BA.2, BA.3, BA.4, BA.5) that have emerged were classified as variants of concern (VOCs) till recently [4,28]. Omicron variants possess unique spike mutations within the receptor-binding domain (RBD), which are key for the evasion of neutralizing antibodies [6,9,29,30]. Omicron BF.7 (BA.5.2.1.7) subvariant has been circulating in numerous countries since mid-2022 [11]. High prevalence of a sublineage of BF.7 (named BF.7.14 by PANGO) with three additional unique mutations (ORF7a: H47Y, NSP2: V94L, and S: C1243F) was reported in China [10]. Nevertheless, ORF7a: H47Y mutation had been detected in isolates from Oceania and North America early in 2020 and in the Omicron BF.5 lineage in Japan at the end of 2022 [11,31]. However, the impact of H47Y mutation on the function of SARS-CoV-2 ORF7a has not been studied.

In this study, we elucidate the impact of H47Y mutation found in the BF.5 and BF.7 sublineages on SARS-CoV-2 ORF7a functions. We show that SARS-CoV-2 ORF7a: H47Y mutation does not affect its ability to counteract the antiviral effect of SERINC5, but inhibits the ability of SARS-CoV-2 ORF7a to antagonize IFN-I response and downregulate MHC-I cell surface levels.

## 2. Results

### 2.1. SARS-CoV-2 ORF7a: H47Y Mutation

**Figure 1:**
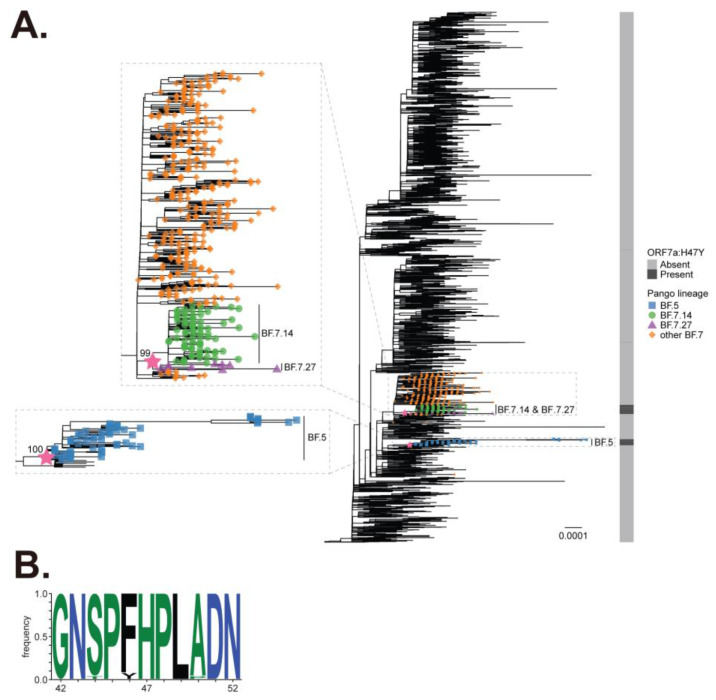
SARS-CoV-2 ORF7a H47Y mutation is prevalent among Omicron sublineages BF.5 and BF.7 and is highly conserved among sarbecoviruses (**A**) A phylogenetic tree of SARS-CoV-2 in the BA.5 lineage. At most, ten genomic sequences per BA.5 subvariant were randomly selected for phylogenetic tree reconstruction using IQ-TREE v2.2.0 software, resulting in 5226 sequences in total. Only BF.5 and BF.7 subvariants are labeled. The ultrafast bootstrap values of the MRCA of BF.5 and that of BF.7.14 and BF.7.27 are 100 and 99, respectively. A star represents the occurrence of ORF7a: H47Y substitution. Only the occurrence of ORF7a: H47Y substitution at an internal node with at least 5 descendant tips harboring the ORF7a:H47Y substitution is shown. (**B**) A sequence logo plot showing an amino acid frequency in ORF7a of 78 sarbecoviruses from position 42 to 52. High conservation of histidine (H) at position 47 of ORF7a in most sarbecoviruses is evident from the sequence logo plot provided.

To trace the occurrence of ORF7a: H47Y substitution along the evolutionary history of the BA.5 lineage, we reconstructed the phylogenetic tree of 5226 SARS-CoV-2 isolates in the BA.5 lineage (Figure 1A) using at most ten randomly selected genomic sequences per each of the 523 BA.5 subvariants and performed the reconstruction of the ancestral state of ORF7a: H47Y substitution. We found two ORF7a: H47Y substitution occurrences in the BA.5 lineage. The first one occurred in the most recent common ancestor (MRCA) of BF.5 subvariants and the second one occurred in the MRCA of BA.7.14 and BF.7.27 subvariants (Figure 1A). In addition, a different mutation in the 47th residue of ORF7a (H47N) was reported in South Korea early in 2020 [32]. It is noteworthy that H47Y mutation was also reported in SARS-CoV ORF7a [33]. Nevertheless, the histidine (H) residue at the 47th amino acid position of ORF7a is conserved in all genomic sequences of 78 sarbecoviruses we analyzed, including those of SARS-CoV, SARS-CoV-related, SARS-CoV-2, and SARS-CoV-2-related viruses [34] (Figure 1B). Sporadic substitutions at this position among SARS-CoV/SARS-CoV-2 viruses indicate that these changes may affect protein functions.

### 2.2. SARS-CoV-2 ORF7a: H47Y Mutation Has No Effect on Its Ability to Counteract SERINC5

**Figure 2:**
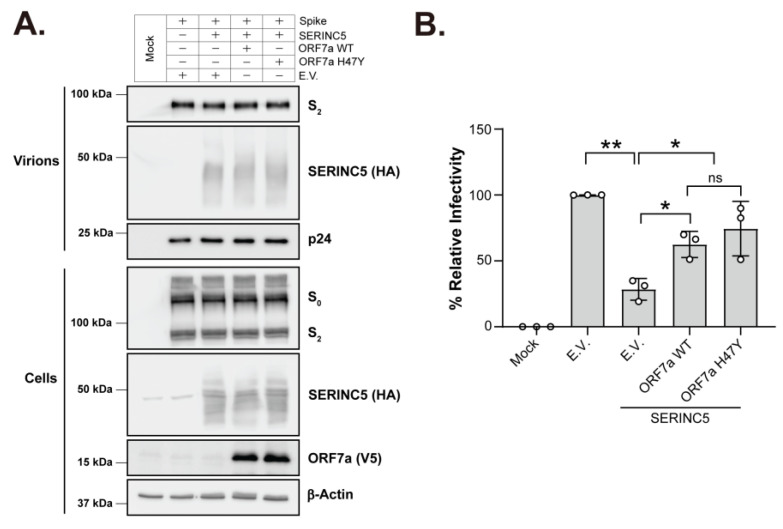
SARS-CoV-2 ORF7a: H47Y mutant retains the anti-SERINC5 activity. (**A**) SARS-CoV-2 ORF7a: H47Y mutation does not affect its steady-state expression or SERINC5 incorporation in viral particles. HEK 293T cells were cotransfected with plasmids for HIV-1NLΔEnv-NanoLuc, SARS-CoV-2 spike, SERINC5, SARS-CoV-2 ORF7a WT/H47Y or empty vector (E.V.) as indicated. Forty-eight hours posttransfection, the indicated proteins were analyzed by immunoblotting in cell lysates and culture supernatants. Representative immunoblot images from 3 independent experiments are shown. (**B**) SARS-CoV-2 ORF7a: H47Y mutant counteracts the antiviral effect of SERINC5. HEK 293T-hACE2 cells were infected with SARS-CoV-2 S pseudovirus from (**A**) and luciferase levels were measured 48 hpi. The percentage of relative infectivity with respect to pseudovirus produced in the presence of E.V. is shown. Results are presented as means ± SD from 3 independent experiments. Statistical comparisons were performed by one-sample *t*-test (two-tailed) between E.V. and SERINC5 + E.V. conditions and unpaired *t*-test (two-tailed) between SERINC5 + E.V. and SERINC5 + ORF7a WT/H47Y conditions. *, *p* < 0.05; **, *p* < 0.01; ns, not significant. (hpi: hours postinfection, S_0_: full-length spike; S_2_: spike S2 subunit).

It was previously shown that SERINC5 becomes incorporated in nascent SARS-CoV-2 virions, resulting in virus entry inhibition by interfering with SARS-CoV-2 S-mediated fusion [13]. We also found that SARS-CoV-2 ORF7a alleviated SERINC5-mediated restriction of viral infectivity by preventing SERINC5 incorporation in nascent virions, as well as forming a complex with S and SERINC5 [13]. To examine the effect of the H47Y mutation of ORF7a on its ability to counteract the antiviral effect of SERINC5 on SARS-CoV-2 entry, we generated SERINC5-containing SARS-CoV-2 S pseudotyped viruses in the presence of either wild-type (WT) or H47Y mutant ORF7a by cotransfecting HEK 293T cells using a replication-defective HIV-1 proviral luciferase reporter plasmid (pHIV-1NLΔEnv-NanoLuc), SARS-CoV-2 S, SERINC5 and either WT or H47Y ORF7a. Initially, we verified that H47Y mutation had no effect on the steady-state expression level of ORF7a protein (Figure 2A). SERINC5, as previously described [13], has a smear-like appearance, probably due to it being a glycosylated protein. We then utilized these pseudoviruses to infect HEK 293T-hACE2 cells, and luciferase levels were measured 48 h postinfection (hpi). As expected, the presence of SERINC5 reduced the infectivity of SARS-CoV-2 S pseudoviruses (Figure 2B). In the case of the ORF7a H47Y mutant, we observed that the mutant ORF7a counteracted the SERINC5 antiviral effect on virion infectivity similarly to WT ORF7a (Figure 2B). Therefore, we conclude that the H-to-Y change at amino acid 47 of ORF7a does not interfere with the ability of ORF7a to counteract the SERINC5 antiviral function.

### 2.3. SARS-CoV-2 ORF7a: H47Y Is Unable to Block the Type I IFN Response

**Figure 3:**
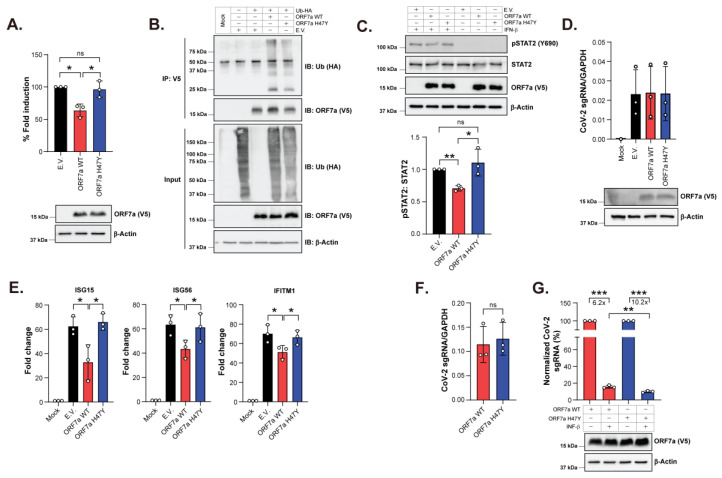
SARS-CoV-2 ORF7a: H47Y mutant is ineffective in antagonizing IFN-I response. (**A**) H47Y mutation abrogates ORF7a’s ability to block IFN-I response. ISG56-promoter driven firefly luciferase reporter levels upon infection with SARS-CoV-2 WT/H47Y or empty vector (E.V.) in the presence or absence of human IFN-β. Data were analyzed by normalizing firefly luciferase to renilla luciferase activities and then normalizing to non-IFN-β-treated samples to obtain fold induction. E.V. was set to 100-fold induction. (**B**) H47Y mutation decreases ORF7a ubiquitination. Lysates of HEK 293T cells cotransfected with plasmids for ubiquitin and either WT, H47Y ORF7a or empty vector (E.V.) followed by immunoprecipitations using an anti-V5 antibody and immunoblot analysis probing for ORF7a (V5), ubiquitin (HA) and β-actin. (**C**) ORF7a H47Y mutant fails to block STAT2 phosphorylation. STAT2 phosphorylation levels were determined by immunoblotting of lysates of HEK 293T cells transfected with plasmids for either SARS-CoV-2 WT, H47Y mutant ORF7a or empty vector (E.V.) and treated with human IFN-β (5 units/mL). Densitometry analysis of phosphorylated STAT2 over total STAT2 levels (pSTAT2: STAT2) in the presence of IFN-β is shown below (E.V. condition is set at 1). (**D**,**E**) H47 mutation impairs ORF7a’s ability to suppress host antiviral response upon SARS-CoV-2 infection. Calu-3 cells transfected with plasmids for SARS-CoV-2 WT/H47Y or empty vector (E.V.) were infected with SARS-CoV-2-eGFP/ΔORF7a virus followed by RT-qPCR for viral subgenomic (sg) RNA levels in (**D**) and expression of the indicated ISGs in (**E**). In (**E**), fold expression changes relative to mock-infected and normalized to GAPDH are shown. (**F**,**G**) SARS-CoV-2 replication in the presence of ORF7a H47Y is severely diminished upon IFN-β treatment. HEK 293T-hACE2 cells transiently expressing either ORF7a WT or H47Y were treated with either IFN-β or mock (PBS) followed by infection with SARS-CoV-2-eGFP/ΔORF7a virus. Viral RNA levels were determined by RT-qPCR and ORF7a expression was analyzed by immunoblotting. In (**F**), SARS-CoV-2 spike copy number normalized to GAPDH in the absence of IFN-β. In (**G**), percentage of normalized SARS-CoV-2 spike copy number was determined after setting mock-treated conditions (for both ORF7a WT and ORF7a H47Y) at 100%. Immunoblot images of ORF7a protein expression are shown. The fold differences between mock- and IFN-β-treated conditions are indicated. Error bars represent means ± S.D for 3 independent experiments. In A, C and G, one sample *t*-test (two-tailed) was used for comparisons between E.V. and ORF7: WT/H47Y conditions, while unpaired *t*-test (two-tailed) was used for comparisons between ORF7a WT and H47Y conditions. Comparisons in E and F were performed using unpaired *t*-test (two-tailed). Representative immunoblot images (*n* = 3) are shown. *, *p* < 0.05; **, *p* ≤ 0.01; ***, *p* ≤ 0.001; ns, not significant.

Previous studies have reported that SARS-CoV-2 ORF7a inhibits type I IFN signaling by targeting STAT2 phosphorylation [15,16]. We utilized an ISG56-promoter-driven luciferase assay to compare the ability of ORF7a WT and H47Y mutant to inhibit the type I IFN response. HEK 293T cells expressing ORF7a WT or H47Y mutant proteins were treated with IFN-β followed by measurement of luciferase levels as a marker of ISG56 promoter activity. In agreement with previous reports, we found that the presence of WT ORF7a significantly inhibited the IFN-β-mediated activation of the ISG56 promoter (Figure 3A). However, ORF7a H47Y mutant had no effect on the ISG56 promoter activity in the presence of IFN-β, similar to what was observed in the presence of an empty vector (E.V.) (Figure 3A). These results show that the H47Y mutation of ORF7a interferes with the ability of ORF7a to inhibit type I IFN signaling.

Because polyubiquitination at residue K119 has been reported to be critical for the ability of ORF7a to antagonize the type I IFN response by means of inhibiting STAT2 phosphorylation [15], we next assessed the effect of H47Y mutation on ORF7a polyubiquitination and interference with STAT2 phosphorylation. To examine ORF7a ubiquitination, we cotransfected HEK 293T cells with plasmids expressing either WT or H47Y mutant ORF7a along with HA-tagged ubiquitin. ORF7a proteins were immunoprecipitated and probed for ubiquitin. We found similar ubiquitinated band patterns, yet a decrease in band intensity, among the WT and H47Y mutant ORF7a proteins (Figure 3B, IP), suggesting that H47Y mutation affects ORF7a ubiquitination. Interestingly, we also repeatedly observed a decrease in the total cellular ubiquitination levels in the presence of ORF7a H47Y mutant when compared to WT ORF7a (Figure 3B, input). Next, to examine whether ORF7a H47Y mutant can suppress STAT2 phosphorylation similarly to WT ORF7a, HEK 293T cells were transfected with either ORF7a WT, ORF7a H47Y or E.V., and stimulated with IFN-β (5 units/mL), followed by immunoblotting probing for STAT2 Y690 phosphorylation. In agreement with previous findings [15], we observed that ORF7a WT reduced IFN-β-mediated STAT2 phosphorylation (Figure 3C). In contrast, ORF7a H47Y mutant did not significantly affect STAT2 phosphorylation, as phosphorylation levels were comparable to those observed in the E.V. condition (Figure 3C). Taken together, our results suggest that H47Y mutation reduces ORF7a polyubiquitination and impairs the ability of ORF7a to inhibit IFN-β-mediated signaling by interfering with STAT2 phosphorylation.

We also examined the effect of ORF7a H47Y mutation on the host antiviral response in the context of SARS-CoV-2 infection. For these studies, we used a recombinant infectious clone of SARS-CoV-2 virus (icSARS-CoV-2-eGFP/ΔORF7a) in which the ORF7a gene has been deleted and replaced with the enhanced green fluorescent protein (eGFP) gene [13,35]. Calu-3 cells, a human pneumocyte cell line, were transfected with plasmids expressing either ORF7a WT, ORF7a H47Y or E.V. followed by infection with ΔORF7a virus (0.5 multiplicity of infection (MOI)) or mock (media only)-infected. Thus, during infection, ORF7a is expressed in trans and a previous report has shown that during SARS-CoV-2 infection, ORF7a expressed in trans acts similarly to ORF7a expressed directly by the virus [13]. Cells were harvested 4 hpi and host and viral RNA levels were determined by quantitative real-time PCR (RT-qPCR), while ORF7a expression was examined by immunoblotting. At first, we verified that the transfected Calu-3 cells were successfully infected, as seen when measuring viral subgenomic RNA (SARS-CoV-2 spike RNA) copy numbers (Figure 3D). In agreement with previous reports [36,37], we found robust stimulation of antiviral genes, including IFN-stimulated gene 15 (ISG15), ISG56, and IFN-induced transmembrane protein 1 (IFITM1) upon SARS-CoV-2 infection (Figure 3E). Interestingly, we observed that when compared to the E.V. condition, the transcript levels of all the aforementioned antiviral genes were decreased in cells expressing ORF7a WT, while expression of the ORF7a H47Y mutant had no effect on RNA levels (Figure 3E). These findings suggest that ORF7a H47Y mutant cannot suppress ISG expression during infection.

Previous reports have shown that IFN-β treatment blocks SARS-CoV-2 replication [36,38,39]. Having known that H47Y mutation interferes with the ability of ORF7a to inhibit type I IFN signaling, we examined the effect of IFN-β treatment on SARS-CoV-2 ΔORF7a virus replication in the presence of either WT or H47Y mutant of ORF7a. For this, we transfected HEK 293T-hACE2 cells with plasmids expressing either ORF7a WT or ORF7a H47Y. Transfected cells were either treated with IFN-β or mock (PBS) for 16 h followed by infection with ΔORF7a virus (0.01 MOI). Cells were collected 24 hpi and viral RNA levels were determined by RT-qPCR, while ORF7a protein levels were determined by immunoblotting. We observed that in the absence of IFN-β, ΔORF7a virus replicated similarly in HEK 293T-hACE2 cells transfected with either WT or H47Y mutant ORF7a (Figure 3F). Upon IFN-β treatment, we observed a decrease in viral transcripts; however, the effect was more severe in cells expressing ORF7a H47Y than those expressing ORF7a WT (Figure 3G). In conclusion, our results suggest that H47Y mutation renders ORF7a unable to antagonize the type I IFN response.

### 2.4. SARS-CoV-2 ORF7a: H47Y Mutation Abrogates Its Potential to Downregulate Surface MHC-I

**Figure 4:**
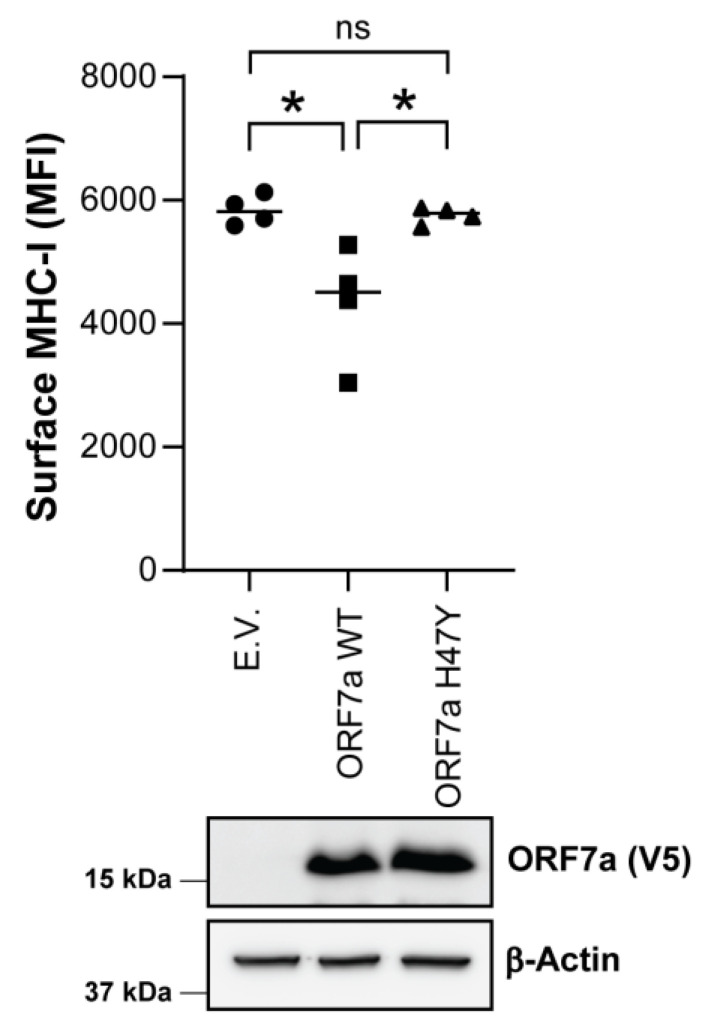
SARS-CoV-2 ORF7a: H47Y mutation impairs its ability to downregulate cell surface MHC-I levels. HEK 293T cells were cotransfected with plasmids for eGFP and ORF7a WT/H47Y or empty vector (E.V.). At 24 h posttransfection, cells were stained with a pan-HLA-ABC antibody (W6/32) conjugated with Alexa Fluor 647 followed by flow cytometry. GFP-positive cells were gated and compared for MHC-I surface levels (median fluorescent intensity). Error bars represent means ± S.D for 4 independent experiments. Statistical comparisons were performed using unpaired *t*-test (two-tailed). Shown below are representative immunoblot images (*n* = 4) for verifying ORF7a protein expression in transfected cells. Cell lysates in transfected cells were harvested at 24 h posttransfection. *, *p* < 0.05; ns, not significant.

Another major function of SARS-CoV-2 ORF7a is to physically interact with and retain MHC-I complexes in the endoplasmic reticulum (ER), preventing their transport to the cell surface [17,18]. Thus, we examined the impact of histidine (H)-to-tyrosine (Y) amino acid substitution at position 47 of ORF7a in preventing the transport of MHC-I to the plasma membrane. We transfected HEK 293T cells with either ORF7a WT, ORF7a H47Y or E.V. and measured MHC-I surface levels 24 h posttransfection by flow cytometry. We observed that surface levels of MHC-I were reduced in cells expressing WT ORF7a when compared to those transfected with E.V. (Figure 4). Interestingly, unlike WT ORF7a, ORF7a H47Y mutant did not alter the MHC-I surface levels (Figure 4). Thus, we conclude that the H47Y mutation in SARS-CoV-2 ORF7a disrupts its ability to downregulate MHC-I surface levels.

### 2.5. The H47Y Mutation in SARS-CoV-2 ORF7a Causes Altered MHC-I Interaction

**Figure 5:**
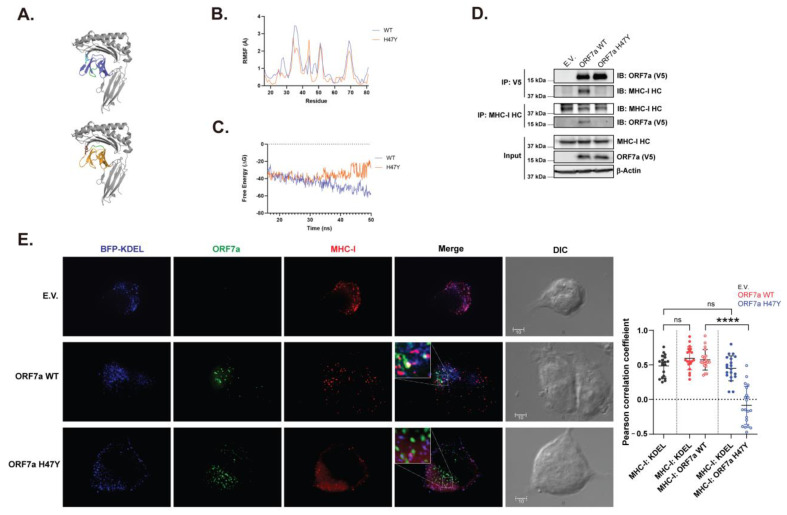
The H47Y mutation in SARS-CoV-2 ORF7a alters its interaction with MHC-I. (**A**–**C**) SARS-CoV-2 H47Y mutation impacts its structural dynamics. (**A**) Representative cartoon models from ClusPro docking simulations of WT (blue) or H47Y mutant (orange) SARS-CoV-2 ORF7a with MHC-I HLA-A2 (gray). Regions highlighted in green represent areas where increased flexibility was observed in the comparison between WT and mutant ORF7a. (**B**) Graphs depicting the root mean square fluctuation (RMSF, angstroms) calculated by CABS-flex 2.0 of the residues of WT and H47Y mutant SARS-CoV-2 ORF7a within the ORF7a–MHC-I complex. (**C**) Graphical representation of the average free energy trajectory values from 3 independent simulations of MHC-I with WT and H47Y mutant SARS-CoV-2 ORF7a. (**D**) SARS-CoV-2 ORF7a H47Y mutant does not physically interact with MHC-I. HEK 293T cells were transfected with SARS-CoV-2 ORF7a WT/H47Y or empty vector. Cells were harvested 48 h posttransfection and lysates were immunoprecipitated with anti-V5 and anti-HLA class I ABC (MHC-I HC) antibodies followed by immunoblot analyses probing with anti-V5 (SARS-CoV-2 ORF7a), anti-MHC-I HC, and anti-β-actin antibodies. Representative immunoblot images are shown. (**E**) SARS-CoV-2 ORF7a H47Y mutant does not colocalize with MHC-I at the ER. AD-293 cells cotransfected with plasmids expressing SARS-CoV-2 ORF7a WT/H47Y and BFP-KDEL were subjected to immunostaining. Representative deconvolved single Z-section images are shown. Scale bar = 10 μm. Insets represent 14.6 × zoomed images from the indicated boxed regions. Graphs on the right show quantitative analyses for colocalization between MHC-I HC, SARS-CoV-2 WT or H47Y mutant and KDEL performed using a region of interest defined by the presence of BFP-KDEL signal with ImageJ (FIJI) Coloc2 plugin. All results are shown for 3 independent experiments. In E, statistical comparisons for MHC-I: KDEL among three conditions were performed using one-way ANOVA followed by Dunnett’s test and comparisons between MHC-I: ORF7a WT and MHC-I: ORF7a H47Y were performed using unpaired *t*-test (two-tailed). ns, non-significant; ****, *p* ≤ 0.0001.

A possible explanation for the inability of the SARS-CoV-2 ORF7a H47Y mutant to attenuate MHC-I levels on the surface of the cell is due to altered interactions with MHC-I. Molecular dynamic simulations have previously been used to describe the SARS-CoV-2 ORF7a–MHC-I interaction [17]. We reasoned that similar simulations could be used to investigate differences in binding to MHC-I between the WT and H47Y mutant ORF7a. We first performed protein–protein docking using ClusPro to model the interaction of WT and H47Y mutant ORF7a with MHC-I HLA-A2 (Figure 5A). We observed that both the WT and the H47Y mutant ORF7a docked to the same region of MHC-I (Figure 5A). We then utilized CABS-flex 2.0 to determine changes in flexibility of both WT and mutant ORF7a bound to MHC-I.

We observed two regions of altered structural flexibility, one of which was more flexible in ORF7a WT (between residues 19 and 23) and one that was more flexible in the ORF7a H47Y mutant (between residues 27 and 30) (Figure 5B). Interestingly, the region affected in the ORF7a H47Y mutant is within the predicted interface of ORF7a with MHC-I, which may alter the protein–protein interaction (Figure 5A). This suggested that the H47Y mutation might compromise the ORF7a–MHC-I interaction. We therefore performed molecular dynamic (MD) simulations using the WebGro server and determined the binding affinity (ΔG) trajectory of WT and H47Y mutant ORF7a in complex with MHC-I. Interestingly, we found that the binding affinity of ORF7a H47Y mutant with MHC-I appeared to be lower than that for the WT ORF7a over the course of MD simulation time (Figure 5C). Together, these data show that the H47Y mutation of SARS-CoV-2 ORF7a may alter the architecture of the interaction between ORF7a and MHC-I, rendering it less efficient. We concluded that the observed changes in protein dynamics may influence the functionality or stability of ORF7a like other single amino acid residue mutations previously identified [24].

To further validate our in silico findings, we performed coimmunoprecipitations (coIPs) and assessed physical interaction between WT and H47Y mutant ORF7a with MHC-I. HEK 293T cells were transfected with plasmids expressing either ORF7a WT, ORF7a H47Y, or E.V., and coIPs were performed using either anti-V5 (ORF7a) or anti-HLA class I ABC (MHC-I heavy chain [HC]) antibodies. We noticed that endogenously expressed MHC-I HC was precipitated when pulling down for WT ORF7a. However, ORF7a H47Y mutant failed to pull down MHC-I HC (Figure 5D). Reciprocal coIPs using anti-HLA class I ABC antibody, in agreement with our aforementioned data, showed that only WT ORF7a, but not ORF7a H47Y mutant precipitated with MHC-I HC (Figure 5D). Our data show that H47Y mutation renders ORF7a unable to interact with MHC-1 HC.

We next examined by immunofluorescence the colocalization pattern of either ORF7a WT or H47Y mutant with MHC-I. We cotransfected AD-293 cells with expression plasmids for either ORF7a WT, ORF7a H47Y, or E.V., along with BFP-KDEL (blue fluorescent protein-tagged lysine–aspartic acid–glutamic acid–leucine) peptide sequence, an ER marker [40]. Transfected cells were fixed 24 h later and stained with anti-V5 (ORF7a) and anti-HLA class I ABC antibody followed by confocal microscopy. We found that WT ORF7a colocalized with MHC-I at the ER, whereas ORF7a H47Y mutant did not (Figure 5E). This is further evident when we measured Pearson correlation coefficients (Figure 5E, graph). However, the colocalization of MHC-I HC to the ER marker KDEL was unaffected across all conditions examined (Figure 5E). In summary, our findings suggest that H47Y mutation affects ORF7a’s ability to interact with MHC-I HC.

## 3. Discussion

Herein, we focused on determining the effect of the ORF7a: H47Y mutation, prevalent among the SARS-CoV-2 Omicron subvariants BF.5 and BF.7, in various known functions of ORF7a, namely, its anti-SERINC5 effect, its ability to antagonize the type I IFN response, and its capacity to downregulate MHC-I cell surface levels.

We found that SARS-CoV-2 ORF7a carrying the H47Y mutation retains its ability to block the SERINC5-mediated restriction of SARS-CoV-2 infectivity. This is in agreement with our previous findings, where we showed that deleting the SARS-CoV-2 ORF7a β-strands including the β-strand D, wherein the 47th amino acid (histidine) lies, has no effect on its anti-SERINC5 function which is governed by its TM domain [13]. We speculate that with an unchanged TM domain, this ORF7a mutant is capable of interacting with SARS-CoV-2 S and SERINC5 similar to the SARS-CoV-2 ORF7a WT protein and hence able to block the antiviral effect of SERINC5.

Unlike SARS-CoV-2 ORF7a WT, our data showed that the H47Y mutation abolished the ability of SARS-CoV-2 ORF7a to suppress the type I IFN response. SARS-CoV-2 ORF7a polyubiquitination of residue K119 has been reported to be critical for interfering with type I IFN response by blocking STAT2 phosphorylation [15]. We found that H47Y mutation reduced ORF7a ubiquitination and affected the ability of ORF7a to suppress STAT2 phosphorylation. Furthermore, our data from SARS-CoV-2 infection experiments showed that H47Y mutation decreased ORF7a’s ability to suppress expression of ISGs (ISG15, ISG56, and IFITM1) and exaggerated IFN-β-mediated blockage of viral replication. Interestingly, we also consistently observed reduced levels of total polyubiquitinated proteins in our coIP inputs from cells expressing ORF7a H47Y (Figure 3B). More studies are needed to further elucidate the mechanism by which ORF7a H47Y mutation interferes with the ubiquitination of other cellular proteins.

We also found that the H47Y substitution in SARS-CoV-2 ORF7a rendered it incapable of downregulating surface MHC-I. Our in silico analysis showed SARS-CoV-2 ORF7a H47Y substitution altered structural flexibility within the predicted interface of ORF7a with MHC-I, likely altering the efficiency of protein–protein interaction. Furthermore, our coIPs along with our immunofluorescence experiments verified that H47Y mutation renders ORF7a incapable of physically interacting and colocalizing with MHC-I. A recent study reported that Phe residue at position 59 (F59) in the E-F loop of SARS-CoV-2 ORF7a protein is a critical determinant for its ability to interact with MHC-I and retain it within the ER [18]. It is interesting to note that the H47Y residue lies along a deep groove formed between the C-D and E-F loops of SARS-CoV-2 ORF7a protein and thus may alter the interactions of ORF7a with MHC-I [12,33]. In fact, it is well established that naturally occurring mutations in viral proteins can have deleterious effects in their anti-MHC-I function. For example, polymorphisms within the HIV-1 accessory protein negative factor (Nef) modulate Nef-induced endocytosis of MHC-I from the cell surface [41]. Moreover, a recent report identified that certain Omicron subvariants are able to downregulate MHC-I more efficiently from the surface of the cell than earlier isolates [42]. It is noteworthy that although the H47Y mutation affects the ability of ORF7a to counteract the type I IFN response and to downregulate MHC-I from the cell surface, SARS-CoV-2 counteracts these two processes utilizing additional viral encoded factors (NSP1, NSP6, NSP13, M, N, ORF3a, ORF6, ORF7a, ORF7b) [15,16,43,44,45]. Thus, ORF7a is not the only protein of SARS-CoV-2 responsible for blocking the type I IFN response and MHC-I surface levels.

## 4. Materials and Methods

### 4.1. Data Mining and Reconstruction of Phylogenetic Tree

Surveillance data of 15,843,705 SARS-CoV-2 isolates were retrieved from the GISAID database on 8 August 2023 (https://www.gisaid.org) [46]. We excluded the data of SARS-CoV-2 isolates that (i) lacked PANGO lineage information; (ii) had been collected after 31 July 2023; (iii) were isolated from non-human hosts; (iv) were sampled from the original passage; and (v) whose genomic sequence was no longer than 28,000 base pairs and contained ≥2% of unknown (N) nucleotides, resulting in data on 1,943,768 SARS-CoV-2 isolates in the BA.5 lineage (EPI SET ID: EPI_SET_230817yu). At most, ten genomic sequences of SARS-CoV-2 in each BA.5 subvariant (EPI SET ID: EPI_SET_230817cf) were randomly sampled and were subsequently aligned to the genomic sequence of Wuhan-Hu-1 SARS-CoV-2 isolate (NC_045512.2) using multiple pairwise alignment implemented in ViralMSA v1.1.24 [47]. Gaps in the alignment were removed automatically using TrimAl v1.4.rev22 with-gappyout mode [48], and the flanking edges of the alignment at positions 1–388 and 29,525–29,713 were trimmed manually. A maximum likelihood-based phylogenetic tree was then reconstructed from the alignment using IQ-TREE v2.2.0 [49]. The best-fit nucleotide substitution model was selected automatically using ModelFinder [50]. Branch support was assessed using ultrafast bootstrap approximation [51] with 1000 bootstrap replicates. We omitted a genomic sequence of Wuhan-Hu-1 from the reconstructed tree and rooted the tree using a genomic sequence of SARS-CoV-2 isolate whose tree distance was closest to the Wuhan-Hu-1 isolate.

The state of having or lacking ORF7a: H47Y substitution was assigned to terminal nodes of the reconstructed tree based on the mutation-calling data from the GISAID database. The reconstruction of ancestral states was then performed using the ace function of the ape R package v.5.7-1 [52] with an equal-rate model. An ancestral node with a posterior probability of having a mutation of at least 0.5 is considered to have the mutation, whereas a node with a posterior probability less than 0.5 is considered to lack the mutation. The occurrence of ORF7a: H47Y substitution was then determined from the state change from lacking mutation in the ancestral node to having mutation in the adjacent descendant node. The reconstructed tree was visualized using the ggtree R package v3.8.2 [53]. All the phylogenetic analyses were aided by R v.4.3.1 [54].

### 4.2. Multiple Sequence Alignment and Generation of Sequence Logo Plot

Genomic sequences of 78 sarbecoviruses, including those of SARS-CoV, SARS-CoV-related, SARS-CoV-2, and SARS-CoV-2-related viruses, were retrieved from the previous phylogenetic study [34]. Each genomic sequence was aligned to each other using MAFFT v7.511 [55] with the G-INS-I mode and 1000 maximum iterations. The coding nucleotide sequence of ORF7a was translated into the protein sequence using JalView v2.11.2.7 [56] according to the standard genetic code. A sequence logo plot for the ORF7a protein sequences was generated using WebLogo web service v3.7.12 [57] in default mode.

### 4.3. Cell Lines and Transfections

HEK 293T cells (ATCC, CRL-3216), HEK 293T-hACE2 (BEI Resources, NIAID, NIH, NR-52511) and AD-293 cells (Agilent) were cultured in Dulbecco’s modified Eagle medium (DMEM; Gibco, Grand Island, NY, USA) with 10% (vol/vol) fetal bovine serum (FBS; Sigma, St. Louis, MO, USA), and 100 mg/mL penicillin and streptomycin (P/S; Gibco) at 37 °C and 5.0% CO_2_. Vero E6 cells (BEI Resources, NIAID, NIH, NR-5258) and Calu-3 cells (ATCC, HTB-55) were maintained in DMEM with 10% FBS, 0.1 mM non-essential amino acids (Gibco), 1 mM sodium pyruvate (Gibco), and 100 mg/mL P/S. All cell lines were detached using either 0.05% or 0.25% (Calu-3) trypsin–EDTA (Gibco) after washing once with phosphate-buffered saline (PBS, Fisher Scientific, Fairlawn, NJ, USA). All transfections were performed using Lipofectamine 3000 (Thermo Fisher Scientific, Invitrogen, Carlsbad, CA, USA) as per the manufacturer’s recommendation.

### 4.4. Plasmids

The pBJ5-SERINC5-HA plasmid was obtained from Heinrich Gottlinger [58]. The HIV-1 NL4-3ΔEnv-NanoLuc and pCMV SARS-CoV-2 SΔ19 were obtained from Paul Bieniasz [59]. The ISG56-Luc plasmid has been previously described and was a kind gift from Raymond Roos [60,61]. Other plasmids used in this study included the pEGFP-N1 (Clonetech), pRL-CMV vector (E226A) acquired from Promega, BFP-KDEL (49150) and HA-ubiquitin (18712) acquired from Addgene. The cloning strategy for generating codon-optimized SARS-CoV-2 ORF7a in pCDNA-V5/His TOPO (Invitrogen) has been described previously [13]. This codon-optimized SARS-CoV-2 ORF7a (WT) in pCDNA-V5/His TOPO plasmid was used as a template to generate SARS-CoV-2 ORF7a: H47Y variants using the Phusion SDM kit (Thermo Fischer Scientific, Vilnius, Lithuania) and the following primers: H47Y_F; 5′-ACAAGTTCGCCTTGACGTG-3′ and H47Y_R; 5′-TGTCTGCAAGAGGGTAGAAGG-3′. The PCR-amplified DNA containing vector backbone (5462 bp) and ORF7a (363 bp) was ligated and grown by transforming One Shot® TOP10 competent cells (Thermo Fisher Scientific, Invitrogen, Carlsbad, CA, USA) followed by DNA sequencing to verify the desired nucleotide changes. Finally, the plasmid DNA encoding ORF7a H47Y was digested using HindIII and XhoI and cloned back into pCDNA-V5/His TOPO backbone.

### 4.5. Pseudovirus Production

HEK 293T cells were seeded in a 6-well plate at a cell density of 0.5 × 10^6^ cells/well. The next day, cells were cotransfected with plasmids for HIV-1NL4-3ΔEnv-NanoLuc (2.5 μg), pCMV SARS-CoV-2 SΔ19 (0.73 μg), pBJ5-SERINC5-HA (0.5 μg) or pCDNA SARS-CoV-2 ORF7a-V5/His (WT/H47Y; 0.75 μg) or empty vector. Twenty-four hours after transfection, culture media were removed and replenished. Cells (see Immunoblotting section) and culture supernatants were harvested 48 h posttransfection, processed as described previously [13], and used for infection experiments.

### 4.6. Pseudovirus Infectivity

For pseudovirus infectivity experiments, HEK 293T-hACE2 cells (2.5 × 10^4^ cells/well) were seeded in a 96-well plate. Cells were infected the next day and lysed at 48 hpi. Luminescence was measured using Nano-Glo luciferase system (Promega) and a Biostack4 (BioTek) luminometer. Infectivity was determined by normalizing the luciferase signals to virus levels as determined by Western blots probing for HIV-1 p24^CA^ on culture supernatants.

### 4.7. Immunoblotting

Cell lysates were prepared as previously described [13,62]. The following antibodies were used for probing the blots: mouse anti-SARS-CoV/SARS-CoV-2 S (GeneTex), mouse anti-V5 (Thermo Fisher Scientific), rabbit anti-HA (Cell Signaling Technology, Danvers, MA, USA), mouse anti-HIV-1 p24 (NIH/AIDS Reagent Program, ARP-4121), monoclonal anti-β-actin (Sigma-Aldrich), HRP-conjugated anti-rabbit IgG (Cell Signaling Technology) and HRP-conjugated anti-mouse IgG (EMD Millipore). Signals were detected using the enhanced chemiluminescence detection kits Clarity and Clarity Max ECL (Bio-Rad, Hercules, CA, USA) followed by quantitation of bands intensities using the ImageJ software V1.53 (National Institutes of Health; https://imagej.nih.gov/ij/).

### 4.8. IFN-I Luciferase Reporter Assay

The IFN-I luciferase reporter assay was performed as described previously with some modifications [15]. Briefly, HEK 293T cells were seeded in a 12-well plate at a cell density of 0.25 × 10^6^ cells/well. The next day, cells were cotransfected with pISG56-Luc (100 ng), pRL-CMV (5 ng), pCDNA SARS-CoV-2 ORF7a-V5/His (WT/H47Y; 2 µg) or empty vector plasmids. At 16 h posttransfection, cells were treated with 1000 units/mL of human IFN-β (PBL assay science) or mock-treated (PBS). At eight hours posttreatment, the cells were assayed for dual-luciferase activities using a Dual-Glo Luciferase Assay System (Promega) and a Biostack4 (BioTek) luminometer. Cells from the duplicate wells of mock-treatment conditions were lysed and processed for immunoblotting for confirming SARS-CoV-2 ORF7a (WT/H47Y) expression (see Immunoblotting section).

### 4.9. Ubiquitination Assay

The ubiquitination assay was performed as before with some modifications [15]. Briefly, HEK 293T cells (0.5 × 10^6^ cells/well) were seeded in duplicate in a 6-well plate. The next day, cells were transfected with pCDNA SARS-CoV-2 ORF7a-V5/His (WT/H47Y; 5 µg) and HA-ubiquitin (500 ng) or empty vector plasmids. At 48 hours posttransfection, cells were washed with cold PBS and lysed in 1× RIPA lysis buffer (300 µL). Clarified lysates (2000 µg) were used for immunoprecipitation using the Dynabeads protein A immunoprecipitation kit (Thermo Fisher Scientific) as per the manufacturer’s instructions. Briefly, 50 µL of beads was preincubated with anti-V5 antibody (1:200) (Thermo Fisher Scientific, R960-25) for 20 min at RT, then washed and incubated with cell lysates overnight at 4 °C. The next day, the beads were washed, eluted, and subjected to immunoblot analyses (see Immunoblotting section). For inputs, 10% of clarified lysates were resolved.

### 4.10. STAT2 Phosphorylation Assay

The STAT2 phosphorylation assay was performed following a previously described protocol with some modifications [15]. Briefly, HEK 293T (0.25 × 10^6^) cells were reverse-transfected with pCDNA SARS-CoV-2 ORF7a-V5/His (WT/H47Y; 3 µg) or empty vector plasmids. The cells were then seeded into a 0.01% poly-L-lysine solution (Sigma Aldrich)-coated plate. Twenty-four hours later, cells were again transfected using the same concentrations of the plasmids mentioned before. Forty-eight hours following the second transfection, cells were treated with 5 units/mL of human IFN-β (PBL assay science) or mock-treated (PBS) and incubated for 15 min at 37 °C. Cells were then washed in 1 mL of cold PBS, lysed in 1× RIPA lysis buffer and analyzed by immunoblotting (see Immunoblotting section). The following antibodies were used for probing STAT2 and phosphorylated STAT2: anti-STAT2 (D9J7L; Cell Signaling) and anti-phospho-STAT2 (Y690) (D3P2P; Cell Signaling). Band intensity was determined using the ImageJ software V1.53 (National Institutes of Health; https://imagej.nih.gov/ij/).

### 4.11. SARS-CoV-2 Virus

The following reagent was obtained through BEI Resources, NIAID, NIH: SARS-related coronavirus 2, isolate USA-WA1/2020 ΔORF7a, recombinant infectious clone with enhanced green fluorescent protein (icSARS-CoV-2-eGFP/ΔORF7a) (NR-54002) [13,35]. Infection experiments using SARS-CoV-2 infectious viruses were performed in a biosafety level 3 laboratory at the University at Buffalo Jacobs School of Medicine and Biomedical Sciences, Buffalo, NY, USA. Viruses were propagated and tittered on Vero E6 cells. All experiments were performed using the early passage (p1) viruses.

### 4.12. SARS-CoV-2 Infection of Calu-3 Cells

Calu-3 cells (0.2 × 10^6^) were reverse-transfected with pCDNA SARS-CoV-2 ORF7a-V5/His (WT/H47Y; 3 µg) or empty vector plasmids. The cells were then seeded into a 0.01% poly-L-lysine solution-coated plate 24-well plate. Twenty-four hours later, cells were either mock-infected (media only) or infected with SARS-CoV-2-eGFP/ΔORF7a at 0.1 MOI and harvested 4 hpi. cDNA was synthesized using the SuperScript III First Strand Synthesis kit (Invitrogen) per manufacturer’s recommendation and RT-qPCR was performed using the Power Up SYBR Green PCR master mix kit (Applied Biosystems) in a CFX384 Touch Real-Time PCR detection system (Bio-Rad). Primers used were: SARS-CoV-2 spike: 5′-CCTACTAAATTAAATGATCTCTGCTTTACT-3′/5′-CAAGCTATAACGCAGCCTGTA-3′, interferon-stimulated gene 56 (ISG56): 5′-GCCTAATTTACAGCAACCATGAG-3′/5′-GGCCTTTCAGGTGTTTCACATA-3′, interferon-stimulated gene 15 (ISG15): 5′-GATCACCCAGAAGATCGGCG-3′/5′-GGATGCTCAGAGGTTCGTCG-3′, interferon induced transmembrane protein 1 (IFITM1): 5′-ACTCCGTGAAGTCTAGGGACA-3′/5′-TGTCACAGAGCCGAATACCAG-3′ and GAPDH: 5′-AACGGGAAGCTTGTCATCAATGGAAA-3′/5′-GCATCAGCAGAGGG GGCAGAG-3′ for normalization. Cells from the duplicate wells were lysed and processed for immunoblotting for confirming SARS-CoV-2 ORF7a (WT/H47Y) expression (see Immunoblotting section).

### 4.13. Interferon Treatment and SARS-CoV-2 Infection of HEK 293T-hACE2 Cells

HEK 293T-hACE2 cells (0.1 × 10^6^) were seeded into a 0.01% poly-L-lysine solution-coated 24-well plate. Next day, cells were transfected with pCDNA SARS-CoV-2 ORF7a-V5/His (WT/H47Y; 1 µg) or empty vector plasmids. Twenty-four hours later, cells were treated with 250 units/mL of human IFN-β (PBL assay science, Piscataway, NJ, USA) or mock-treated (PBS). At sixteen hours posttreatment, cells were either mock-infected or infected with SARS-CoV-2-eGFP/ΔORF7a at 0.01 MOI. Cells were harvested 24 hpi followed by RNA isolation, cDNA synthesis and RT-qPCR, as described above (see SARS-CoV-2 infection of Calu-3 cells section). Cells from the duplicate wells were lysed and processed for immunoblotting for confirming SARS-CoV-2 ORF7a (WT/H47Y) expression (see Immunoblotting section).

### 4.14. Cell Surface MHC-I Downregulation Assay

SARS-CoV-2 ORF7a-mediated downregulation of cell surface MHC-I levels was determined by flow cytometry as previously described with some modifications [17,18]. HEK 293T cells were seeded in a 12-well plate at a density of 0.25 × 10^6^ cells/well. The next day, cells were cotransfected with pEGFP-N1 (50 ng), pCDNA SARS-CoV-2 ORF7a-V5/His (WT/H47Y; 1 µg) or empty vector plasmids. At 24 hours posttransfection, cells were detached from the plate and stained with anti-human HLA A, B, C-Alexa Flour 647 W6/32 (BioLegend, San Diego, CA, USA) for 30 min at 4 °C. Cells were washed twice with FACS buffer (PBS containing 2% FBS and 0.8 mM EDTA), fixed with 2% paraformaldehyde for 10 min at 4 °C, and acquired on BD LSRFortessa followed by analysis using FlowJo version 10.8.0. Cells from the duplicate wells were lysed and processed for immunoblotting for confirming SARS-CoV-2 ORF7a (WT/H47Y) expression (see Immunoblotting section).

### 4.15. Protein–Protein Docking and Molecular Dynamic Simulations

Protein docking was performed similarly to methods previously described [17]. Briefly, the crystal structures of SARS-CoV-2 ORF7a (PDBID: 6W37, residues 16–81; resolution: 2.90 Å) [63] and MHC-I HLA-A2 (PDBID: 1DUY; resolution: 2.15 Å) [64] were obtained from the Protein Data Bank (PDB). The H47Y mutation on SARS-CoV-2 ORF7a was performed using the mutagenesis function in the PyMOL Molecular Graphics System, Version 2.5, Schrödinger, LLC. The structures of wild-type and H47Y-mutant ORF7a with MHC-I were then submitted to ClusPro for docking simulations [65,66,67]. To determine alterations in protein flexibility (root mean square fluctuation, RMSF), the docked structures were entered into a CABS-flex 2.0 webserver using default conditions [68]. Molecular dynamic simulations for ORF7a binding affinities were performed using WebGro [69]. Simulations (50 ns with 1000 individual frames each) were run using a CHARMM 27 force field at 300 Kelvin, 1.0 bar of pressure, and at 0.15 M NaCl. Change in free energy states were acquired for every 2 frames from 3 replicate simulations using gmx_MMPBSA [70,71].

### 4.16. Coimmunoprecipitations (coIPs)

HEK 293T cells were seeded in a 10 cm culture dish (3.5 × 10^6^ cells) and transfected with pCDNA SARS-CoV-2 ORF7a-V5/His (WT/H47Y; 7 µg) or empty vector plasmids. At 48 hours posttransfection, cells were washed in cold PBS and lysed in 700 µL of NP-40 lysis buffer containing 5% glycerol and 1× Halt Protease and phosphatase inhibitors (Thermo Fischer Scientific) for 30 min at 4 °C. Lysates were clarified by centrifugation and used for immunoprecipitation using the Dynabeads protein A immunoprecipitation kit (Thermo Fisher Scientific). Dynabeads (30 μL) were preincubated with either mouse anti-V5 (1:200) (Thermo Fisher Scientific, R960-25) or rabbit anti-HLA class I ABC (1: 200) (Proteintech, 15240-1-AP, Rosemont, IL, USA) antibodies for 20 min at RT. Cell lysates (2000 μg) were incubated with antibody-coated Dynabeads followed by overnight incubation at 4 °C. The next day, Dynabeads were then washed and eluted. The eluted fractions and 10% clarified lysates (inputs) were subjected to Western blot analysis (see Immunoblotting section).

### 4.17. Immunofluorescence

Samples were processed for immunofluorescence as previously described [13]. AD-293 cells (5 × 10^4^ cells/well) were seeded on poly-L-lysine-treated 12-mm coverslips (Carolina). The next day, cells were cotransfected with BFP-KDEL (50 ng) and pCDNA SARS-CoV-2 ORF7a-V5/His (WT/H47Y; 500 ng) or empty vector plasmids. At 24 hours posttransfection, cells were washed, fixed with 4% paraformaldehyde and permeabilized with 0.3% Triton X-100 (Fischer Scientific) for 5 min at RT. Cells were then blocked with blocking buffer (1× PBS containing 4% bovine serum albumin [Research Products International] and 0.075% Tween 20 [Research Products International]) for 1 h at RT followed by incubation with mouse anti-V5 (SARS-CoV-2 ORF7a) (1: 300 dilution) and rabbit anti-HLA class I ABC (1:200) (Proteintech, 15240-1-AP) in blocking buffer overnight at 4 °C. Cells were then stained with Alexa Fluor 594 chicken anti-rabbit IgG (1:1500 dilution; Invitrogen) and Alexa Fluor 488 goat anti-mouse IgG (1:1500 dilution; Invitrogen) in blocking buffer for 1 h at RT, washed 3 times in 1× PBS and mounted in antifade mounting media (0.25% 1,4-phenylenediamine and 90% glycerol in 1× PBS). A Z-series of images was acquired using a 100×/1.46 Plan Apo oil immersion objective on a motorized Zeiss Axioimager M2 microscope equipped with an Orca ER charge-coupled-device (CCD) camera (Hamamatsu, Bridgewater, NJ, USA), processed using Volocity (version 6.1, Acquisition Module [Improvision Inc., Lexington, MA, USA]), and deconvolved by a constrained iterative algorithm using the Volocity Restoration Module. Colocalization analyses were performed using a region of interest defined by the presence of the BFP-KDEL signal with ImageJ (FIJI) Coloc2 plugin.

### 4.18. Statistical Analysis

Statistical analyses were performed using GraphPad Prism software version 9.5.0. Statistical tests used to determine significance are described in the figure legends. Comparisons yielding p values less than 0.05 were considered to be significant.

## 5. Conclusions

The present study shows that the H47Y mutation of SARS-CoV-2 ORF7a impairs its ability to antagonize the type I IFN response and to downregulate MHC-I cell surface levels, but has no effect in its anti-SERINC5 function. In conclusion, this study shows that the BF.7- and BF.5-associated ORF7a H47Y mutations of SARS-CoV-2 ORF7a can affect important functions of this SARS-CoV-2 accessory protein, indicating that impairment of these effects of ORF7a may contribute to differences in viral pathogenesis as well as a potential reason behind these strains not spreading efficiently globally.

## Data Availability

All uncropped immunoblot images are included in the Supplementary Materials. Surveillance datasets of SARS-CoV-2 isolates are available from the GISAID database (https://www.gisaid.org; accessed on 8 August 2023; EPI_SET_230817yu, and EPI_SET_230817cf). The supplemental table for each GISAID dataset is available in the GitHub repository (https://github.com/TheSatoLab/ORF7a_H47Y).

## Supplementary Materials

The following supporting information can be downloaded at https://www.mdpi.com/article/10.3390/ijms25042351/s1.
