# Supplementary material for: SARS-CoV-2 ORF7a Mutation Found in BF.5 and BF.7 Sublineages Impacts Its Functions
Source: Int J Mol Sci. 2024 Feb 16;25(4):2351. doi: 10.3390/ijms25042351 (PMC10889720; doi:10.3390/ijms25042351)
Supplement: Supplementary file 1 [file ijms-25-02351-s001.zip › ijms-2865204-supplementary.pdf]

Supplementary Figures

SARS-CoV-2 ORF7a Mutation Found in BF.5 and BF.7 Sublineages Impacts Its Functions

Uddhav Timilsina [1], Emily B. Ivey [1], Sean Duffy [1], Arnon Plianchaisuk [2], The Genotype to Phenotype Japan (G2P-Japan) Consortium, Jumpei Ito 2, Kei Sato [2,3,4,5,6,7,8] and Spyridon Stavrou[1,*]

[1] Department of Microbiology and Immunology, Jacobs School of Medicine and Biomedical Sciences, University at Buffalo, NY, United States
[2] Division of Systems Virology, Department of Microbiology and Immunology, The Institute of Medical Science, The University of Tokyo, Tokyo, Japan
[3] Graduate School of Medicine, The University of Tokyo, Tokyo, Japan
[4] International Research Center for Infectious Diseases, The Institute of Medical Science, The University of Tokyo, Tokyo, Japan
[5] Graduate School of Frontier Sciences, The University of Tokyo, Kashiwa, Japan
[6] International Vaccine Design Center, The Institute of Medical Science, The University of Tokyo, Tokyo, Japan
[7] Collaboration Unit for Infection, Joint Research Center for Human Retrovirus infection, Kumamoto University, Kumamoto, Japan
[8] CREST, Japan Science and Technology Agency, Kawaguchi, Japan
* Correspondence: stavrou2@buffalo.edu

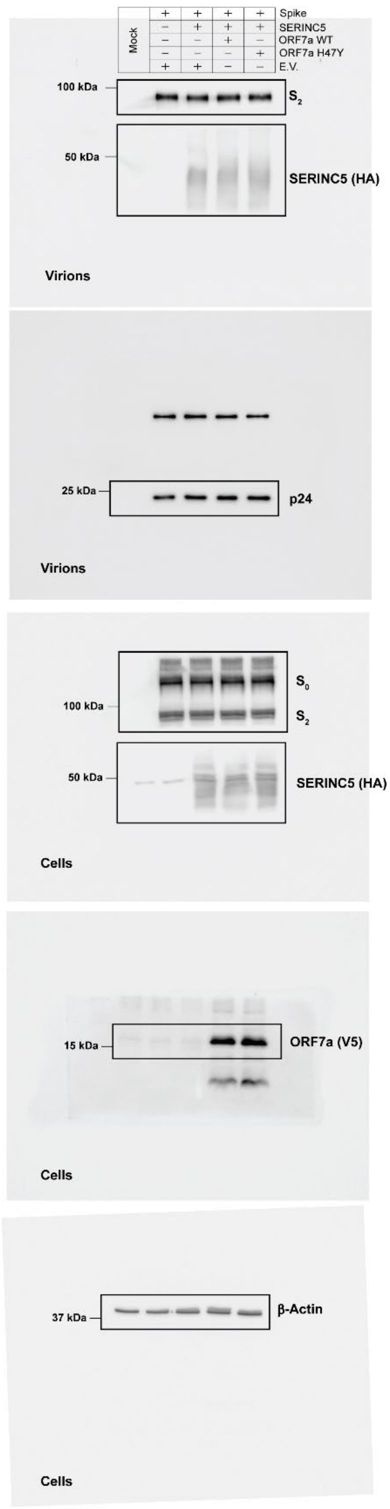

Figure S1: Uncropped immunoblot images for Figure 2.

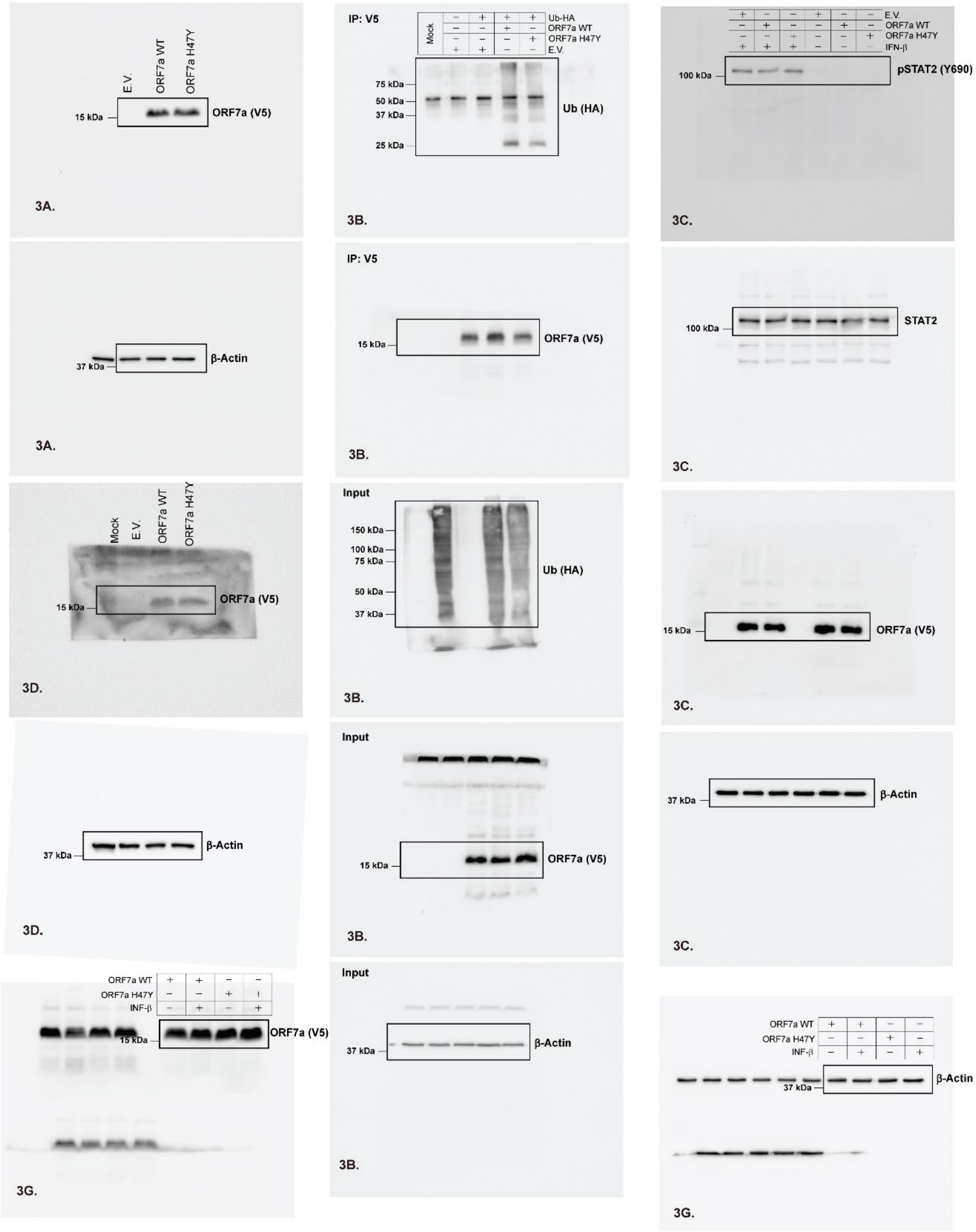

Figure S2: Uncropped immunoblot images for Figure 3.

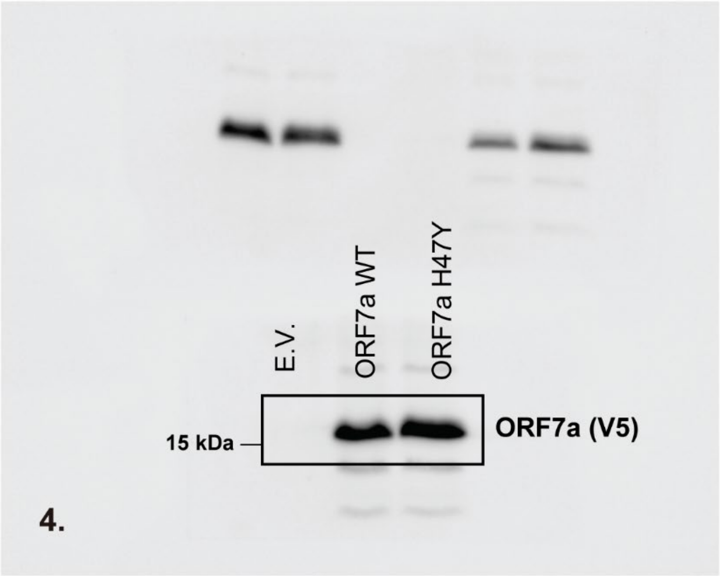

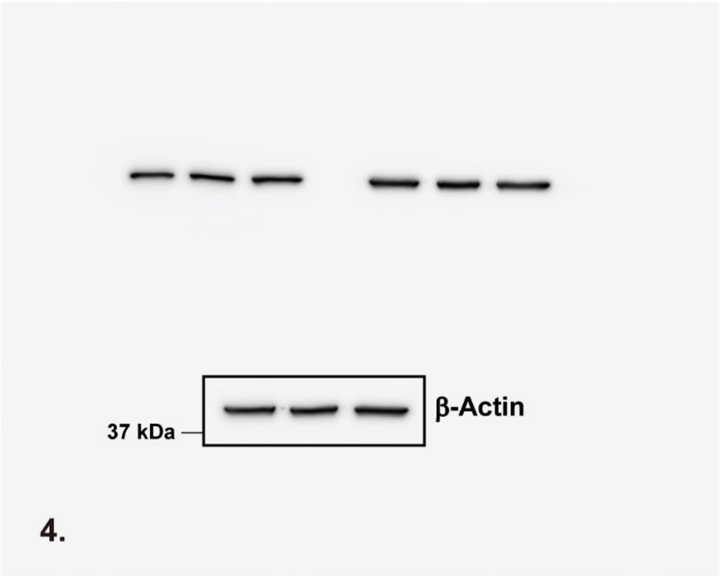

Figure S3: Uncropped immunoblot images for Figure 4.

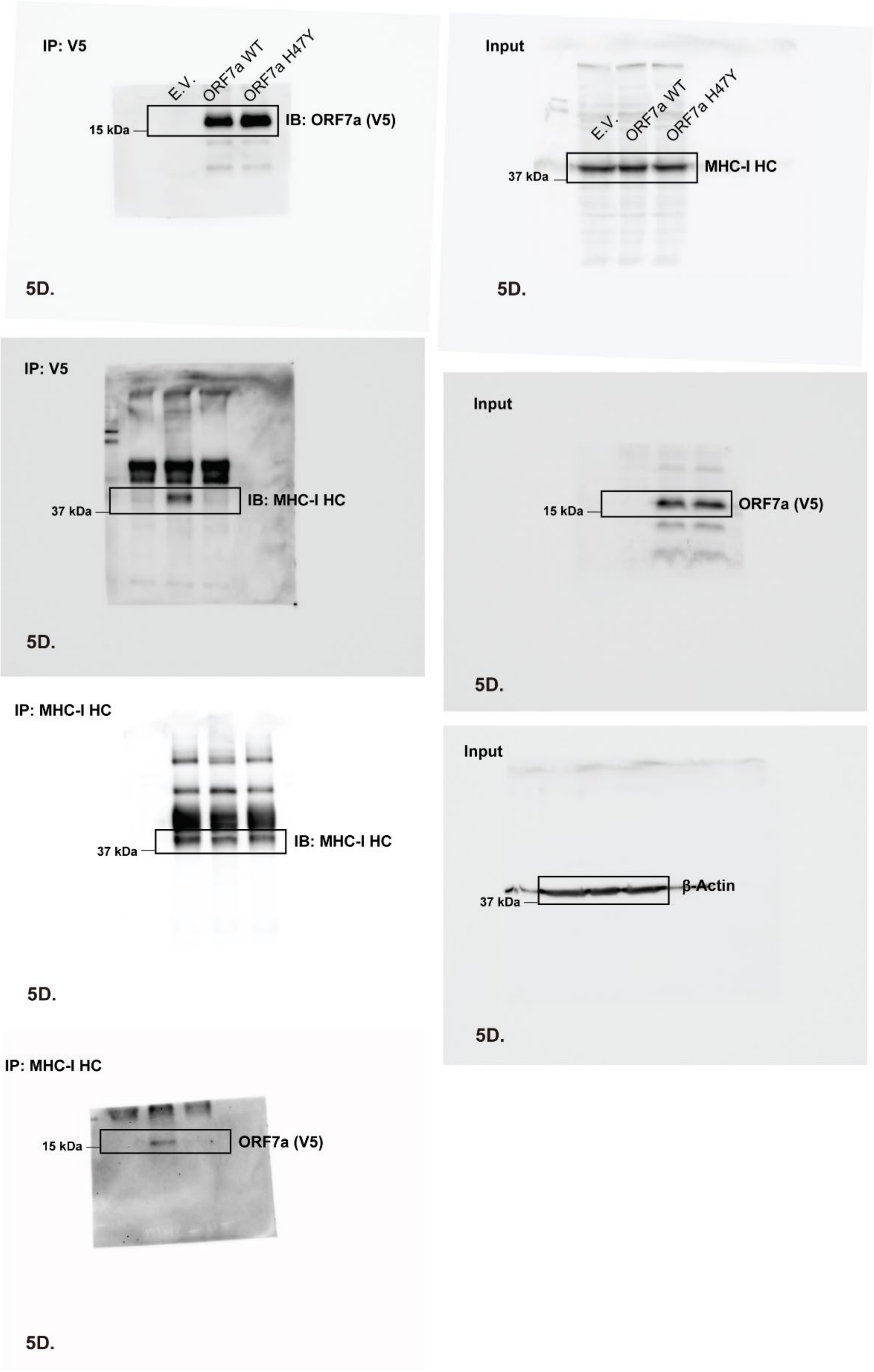

Figure S4: Uncropped immunoblot images for Figure 5.
